# The use of superficial heat in the management of low back pain by chiropractors and osteopaths in Australia: an observational cross-sectional survey

**DOI:** 10.1186/s12998-026-00648-w

**Published:** 2026-06-01

**Authors:** Peter J. McCann, Peter J. McGlynn, Andrew L. Vitiello, Matthew Fernandez, Katie de Luca

**Affiliations:** 1 Chiropractic Course, School of Health, Medical and Applied Sciences, CQUniversity, Brisbane, Australia; 2 Private clinical practice, Williamstown, VIC Australia; 3 Private clinical practice, Sydney, NSW Australia

**Keywords:** Low back pain, Superficial heat, Chiropractor, Osteopath, Clinical practice guideline, Non-pharmacological

## Abstract

**Background:**

Low back pain (LBP) is the leading cause of years lived with disability globally and a major contributor to disease burden in Australia. Superficial heat is recognised as a non-pharmacological intervention for LBP. However, its clinical use among chiropractors and osteopaths remains unclear.

**Objective:**

To examine how chiropractors and osteopaths in Australia use superficial heat in the management of LBP, explore their perceptions of its role and effectiveness, and assess their awareness and application of clinical practice guidelines for LBP.

**Methods:**

A cross-sectional survey was conducted between June and October 2022 among registered chiropractors and osteopaths in Australia. The online 43-item questionnaire captured demographic, practice, and clinical data. This included frequency and method of superficial heat use, perceived effectiveness, and awareness of clinical practice guidelines for LBP. Descriptive statistics were used to summarise findings.

**Results:**

A total of 341 practitioners completed the survey (78.3% chiropractors, 21.7% osteopaths). This represents approximately 3.5% of the total registered chiropractic and osteopathic workforce in Australia. Approximately half of all respondents (44.6%) reported using superficial heat for LBP in the preceding 12 months. Among those, chiropractors were more likely to use superficial heat for chronic LBP (44.6%) than for acute LBP (13.8%), whereas osteopaths were more likely to use it for acute LBP (20.3%). Superficial heat was acknowledged by 71.3% of respondents to have a role in LBP management, while 66.0% regarded it as an adjunct to spinal manipulation. In contrast, superficial heat was recommended for self-management by 34.1% of chiropractors and 28.4% of osteopaths. Awareness of LBP clinical guidelines was moderate, with 58.4% of practitioners reporting they knew how and where to access them. Chiropractors most commonly referred to the American College of Physicians (ACP) guidelines, whereas osteopaths more frequently used the UK NICE guidelines.

**Conclusion:**

Despite its recognised clinical value, the use of superficial heat for LBP was limited, with 44.6% of practitioners reporting its use in practice, and fewer chiropractors (34.1%) and osteopaths (28.4%) recommending it for patient self-management. These findings indicate a gap between awareness and guideline-concordant care, highlighting the need for targeted education and clearer clinical guidance to support its consistent integration within multimodal LBP management.

**Supplementary Information:**

The online version contains supplementary material available at 10.1186/s12998-026-00648-w.

## Introduction

Low back pain (LBP) is the leading cause of years lived with disability (YLDs) worldwide and a major contributor to the global burden of disease [1–5]. In Australia, musculoskeletal conditions are the second largest contributor to the national burden of disease after cardiovascular disease [6, 7], where LBP is estimated to account for approximately 298,624 YLDs, representing 20.1% of the total burden attributed to musculoskeletal disorders [6].

The chiropractic and osteopathic professions have historically emphasised non-pharmacological approaches in the management of musculoskeletal conditions, including LBP [8–11]. Recent updates to clinical practice guidelines for LBP prioritise non-pharmacological strategies as the first-line management of LBP, particularly for chronic LBP [12]. First-line interventions include spinal manipulation, advice to stay active, patient education, reassurance and the use of superficial heat and/or cold [13–15]. Historically, clinical practice guidelines prioritised pharmacological options such as paracetamol for the initial management of LBP [16–18] and did not endorse non-pharmacological interventions as first-line treatments for LBP.

Superficial heat is an acknowledged non-pharmacological intervention in the management of LBP [19–22]. A Cochrane systematic review by French et al. demonstrated that superficial heat moderately reduces pain and disability [20, 21]. A recent narrative review by Freiwald et al. concluded that low-level superficial heat was an effective, safe, and cost-effective intervention for relieving LBP [23]. Superficial heat may be applied as a standalone intervention or in combination with other treatment modalities, such as education, manual therapies and exercise, to improve outcomes in patients with LBP [24, 25]. Its use as an adjunct to manual therapies is commonplace among healthcare practitioners, including physiotherapists, chiropractors, and osteopaths [25]. Methods of application of superficial heat typically involve conduction and convection (heat wraps, wheat bags) [26]. These differ from conversion modalities (ultrasound, low-level laser or diathermy) [27].

While many clinical practice guidelines for LBP endorse superficial heat, the practical guidance regarding its application, frequency and dosage across different presentations of LBP remains limited [14]. The heterogeneity of LBP, variability in the clinical use of superficial heat and differing professional perspectives of its therapeutic value contribute to inconsistency in clinical practice [5]. The application of superficial heat for acute and chronic low back pain varies across clinical contexts, and in the setting of limited and heterogeneous evidence, its use is likely influenced by clinical judgement and practice-based experience [25]. A lack of high-quality studies and potential methodological biases within the existing evidence base further compound these challenges. Additionally, conflicting recommendations across clinical practice guidelines make it difficult to establish consistent conclusions about its clinical effectiveness [21, 28].

To date, there has been no in-depth analysis of chiropractors’ and osteopaths’ use of superficial heat in the management of LBP [29]. Within the Australian chiropractic profession, fewer than 20% of practitioners report frequent use of superficial heat [30]. Similarly, its use within the osteopathic profession appears to be either underutilised or underreported [31]. Previous workforce surveys of chiropractors and osteopaths in Australia [30, 31] have provided insights into practitioner demographics and clinical patterns; however, these studies were not designed to examine the use of superficial heat for LBP specifically. Consequently, there exists limited evidence describing how superficial heat is applied in the management of LBP across these professions. This study aimed to address this gap and provide the first national snapshot of the use of superficial heat for the management of LBP by chiropractors and osteopaths in Australia to explore their patterns of use, perceptions of effectiveness and awareness of relevant clinical practice guidelines for LBP.

## Methods

### Study design

A pragmatist paradigm informed the design of this study and in the interpretation of results, allowing the consideration of multiple practitioner perspectives on the use of superficial heat in the management of LBP. We conducted a cross-sectional survey to capture a point-in-time estimate of reported use of superficial heat by registered chiropractors and osteopaths in Australia. Reporting of this study design follows the STROBE guidelines for cross-sectional studies [32]. (Appendix 1) Ethics approval was granted from the Human Research Ethics Committee (HREC) research division at CQUniversity on October 25th, 2021 (HREC number 0000022481).

### Population and sample

Chiropractors and osteopaths in Australia are nationally registered and regulated health professionals. The target population was chiropractors and osteopaths currently registered with the Australian Health Practitioner Regulation Agency (AHPRA) [33]. As of May 2024, there were 6,345 registered chiropractors and 3,325 osteopaths. The sample included 4.2% of registered chiropractors and 2.2% of registered osteopaths. This represents approximately 3.5% of the total registered chiropractic and osteopathic workforce in Australia. Eligibility required current AHPRA registration for clinical practice in Australia. There were no exclusion criteria applicable to this sample.

### Recruitment

Participants were approached via electronic communication invitations through the national membership databases of three relevant professional associations: the Australian Chiropractors Association (ACA), Chiropractic Australia (CA), and Osteopathy Australia (OA). The general populations of the chiropractic and osteopathic professions in May 2024 were 6,345 and 3,325 registered chiropractors and osteopaths, respectively [33]. A hyperlink to an online survey was sent. Additional recruitment strategies included a flyer at the 2022 ACA and CA annual conferences, a pre-recorded video invitation, Facebook posts and electronic communications from Victoria University’s research division with a QR code link to the survey. Participant recruitment for the online survey took place from June 22, 2022, to October 31, 2022.

### Informed consent and data security

Upon accessing the QR code survey link, participants were provided with study information and then an electronic informed consent page for signing, before accessing the survey. All survey responses were anonymous; IP addresses and duplicate submissions were blocked via Qualtrics settings. Data was stored on secure institutional servers with restricted access. No incentive to participate in the online survey was provided.

### Data collection

Data was collected online, using Qualtrics software, Version 29 (Copyright 2020 Qualtrics) [34]. No validated instrument existed that simultaneously captured practitioners’ frequency and methods of superficial heat application, their perceptions of its effectiveness, and their awareness of clinical practice guidelines for LBP. Therefore, an online survey comprising a 43-item questionnaire tailored to the research questions was developed. Before distribution of the online survey, we conducted a pilot survey with ten registered practitioners (five chiropractors and five osteopaths) to assess face validity, clarity and content coverage [35]. Each practitioner had a minimum of five years of clinical practice experience. Their feedback prompted minor edits to the grammar and wording of the survey questionnaire.

### Practitioner and practice variables

The survey collected data on practitioner characteristics, which included age range, gender, year of registration, years in clinical practice, number of practices, hours worked per week, graduating university, professional association membership, highest qualification, and additional healthcare qualifications. Practice characteristics included practice location classified by the Rural, Remote and Metropolitan Areas (RRMA) system, state location of primary practice, and the number of practitioners at the primary place of practice.

### Superficial heat variables

The survey explored practitioners’ perceptions and use of superficial heat in the management of different presentations of LBP. The primary outcome of the study was self-reported use of superficial heat for different presentations of LBP (any use versus no use). The specific survey items used to assess use of superficial heat for the management of LBP by chiropractors and osteopaths in this study were: “*In what percentage of your acute / sub-acute / chronic low back clients do you use superficial heat application when managing their low back pain?*” Secondary outcomes included frequency of use of superficial heat for different presentations of LBP, methods of application of superficial heat, proportion of cases that were provided with superficial heat for LBP, and advice for patient self-management. Other variables captured included the perceived role and effectiveness of superficial heat, and chiropractors’ and osteopaths’ recommendations for patient self-management. A Likert scale was used for most survey items (1 = Strongly disagree to 5 = Strongly agree). Participants were also asked about their use of superficial heat as a primary therapeutic modality in situations where spinal manipulation was contraindicated or deemed inappropriate.

### Clinical practice guidelines

Survey items asking about clinical practice guidelines were designed to assess practitioner attitudes towards, and awareness of, recommendations of the application of superficial heat in the management of LBP. The specific survey items used to assess attitudes and awareness included: “*Published treatment guidelines recommending the application of superficial heat in the management of LBP are important*”, “*Published treatment guidelines play a role in my clinical decision-making in whether to apply superficial heat application in the management of LBP*” and “*Published treatment guidelines directing the method of superficial heat application in the management of LBP are important*”. A Likert scale was used for these survey items (1 = Strongly disagree to 5 = Strongly agree).

### Statistical analysis

The descriptive characteristics of the analytic sample were determined by the unweighted survey sample size and as a weighted sample percentage, representing the population percentage. Descriptive statistics for each survey item were reported as frequency distributions (counts and proportions). Descriptive statistics summarised practitioner, practice and patient characteristics, practitioner perceptions of the use of superficial heat in the management of LBP and the level of awareness, knowledge, and use of clinical practice guidelines for LBP. All statistical analyses were conducted using the Statistical Package for Social Sciences (SPSS) software [36].

## Results

A total of 341 practitioners completed the survey, comprising 267 chiropractors (78.3%) and 74 osteopaths (21.7%). This corresponded to 4.2% of registered chiropractors and 2.2% of registered osteopaths, representing 3.5% of the combined workforce. Practitioner demographics are summarised in Table 1. Overall, 57.8% of respondents identified as male, with a higher proportion among chiropractors (63.3%) compared to osteopaths (37.8%). The most common age group among respondents across the sample was 31–40 years (29.9%). RMIT University was the most frequently reported graduating institution (39.9%). The most common duration in practice was 11–20 years (31.3%), with similar distributions between professions.

**Table 1:** Sociodemographics of the total sample and for chiropractors and osteopaths

Practitioner characteristics			
Variable	Total sample *n* = 341	Chiropractors *n* = 267	Osteopaths *n* = 74
Sex	Case no. (%)		
Female	144 (42.2%)	98 (36.7%)	46 (62.2%)
Male	197 (57.8%)	169 (63.3%)	28 (37.8%)
Age category			
20–30 years of age	46 (13.5%)	30 (11.2%)	16 (21.6%)
31–40 years of age	102 (29.9%)	83 (31.1%)	19 (25.7%)
41–50 years of age	83 (24.3%)	57 (21.3%)	26 (35.1%)
51–60 years of age	73 (21.4%)	61 (22.8%)	12 (16.2%)
61–70 years of age	32 (9.4%)	32 (12.0%)	0 (0.0%)
> 70 years of age	5 (1.5%)	4 (1.5%)	1 (1.4%)
Years in clinical practice			
0–2 years	34 (10.0%)	23 (8.6%)	11 (14.9%)
3–5 years	30 (8.8%)	21 (7.9%)	9 (12.2%)
6–10 years	48 (14.1%)	36 (13.5%)	12 (16.2%)
11–20 years	106 (31.1%)	81 (30.3%)	25 (33.8%)
21–30 years	64 (18.8%)	51 (19.1%)	13 (17.6%)
> 30 years	59 (17.3%)	55 (20.6%)	4 (5.4%)
Year of registration			
1975–1985	32 (9.4%)	31 (11.6%)	1 (1.3%)
1986–1996	57 (16.7%)	49 (18.3%)	8 (10.8%)
1997–2007	88 (25.8%)	65 (24.3%)	23 (31.1%)
2008–2018	117 (34.3%)	89 (33.3%)	28 (37.8%)
2019–2022	47 (13.8%)	33 (12.4%)	14 (18.9%)
Clinical hours worked per week			
0 h	4 (1.2%)	3 (1.1%)	1 (1.4%)
1–10 h	20 (5.9%)	17 (6.4%)	3 (4.1%)
11–20 h	65 (19.1%)	51 (19.1%)	14 (18.9%)
21–30 h	108 (31.7%)	84 (31.5%)	24 (32.4%)
31–40 h	121(35.5%)	96 (36.0%)	25 (33.8%)
> 40 h	23 (6.7%)	16 (6.0%)	7 (9.5%)
Professional association membership			
Yes	318 (93.3%)	247 (92.5%)	71 (95.9%)
No	23 (6.7%)	20 (7.5%)	3 (4.1%)
Professional association membership type (*n* = 317)			
ACA	140 (41.1%)	140 (52.4%)	0 (0.0%)
ACA + CA/COCA	17 (5.0%)	17 (6.4%)	0 (0.0%)
ACA + Other	6 (1.8%)	6 (2.2%)	0 (0.0%)
CA/COCA	82 (24.0%)	82 (30.7%)	0 (0.0%)
CA/COCA + Other	1 (0.3%)	1 (0.4%)	0 (0.0%)
OA	71 (20.8%)	0 (0.0%)	71 (95.9%)
No response	24 (7.0%)	21 (7.9%)	3 (4.1%)
Additional qualifications			
Yes	82 (24.0%)	71 (26.6%)	11 (14.9%)
No	258 (75.7%)	195 (73.0%)	63 (85.1%)
No response	1 (0.3%)	1 (0.4%)	0 (0.0%)
Additional qualification type			
Grad diploma	27 (7.9%)	24 (9.0%)	3 (4.0%)
Bachelor degree	23 (6.7%)	19 (7.1%)	4 (5.4%)
Masters degree	22 (6.5%)	20 (7.5%)	2 (2.7%)
Doctoral degree	7 (2.1%)	6 (2.2%)	1 (1.3%)
Graduate institute			
CQUniversity	14 (4.1%)	14 (5.2%)	0 (0.0%)
Macquarie University	93 (27.3%)	93 (34.8%)	0 (0.0%)
Murdoch University	24 (7.0%)	24 (9.0%)	0 (0.0%)
RMIT University	136 (39.9%)	108 (40.4%)	28 (37.8%)
Southern Cross University	6 (1.8%)	0 (0.0%)	6 (8.1%)
Victoria University	18 (5.3%)	0 (0.0%)	18 (24.3%)
Other	50 (14.7%)	28 (10.5%)	22 (29.7%)

ACA-Australian Chiropractors Association, CA-Chiropractic Australia, COCA-Chiropractic and Osteopathic College of Australasia, Osteopathy Australia (OA)

Practice characteristics of chiropractors and osteopaths are summarised in Table 2. Most respondents (76.0%) reported practising in cities or metropolitan regions as classified by the Rural, Remote and Metropolitan Areas (RRMA) framework. Victoria was the most common state of primary practice (34.0%), followed by New South Wales (28.7%).

**Table 2:** Practice demographics of the total sample and for chiropractors and osteopaths

Practice demographics			
	Total sample *n* = 341	Chiropractors *n* = 267	Osteopaths *n* = 74
Practice state location			
ACT	9 (2.6%)	7 (2.6%)	2 (2.7%)
NSW	98 (28.7%)	83 (31.1%)	15 (20.3%)
NT	2 (0.6%)	2 (0.7%)	0 (0.0%)
QLD	61 (17.9%)	49 (18.4%)	12 (16.2%)
SA	20 (5.9%)	18 (6.7%)	2 (2.7%)
TAS	9 (2.6%)	7 (2.6%)	2 (2.7%)
VIC	116 (34.0%)	78 (29.2%)	38 (51.4%)
WA	26 (7.6%)	23 (8.6%)	3 (4.1%)
No response	34 (10.0%)	26 (9.7%)	0 (0.0%)
Number of practice locations			
0	7 (2.0%)	6 (2.2%)	1 (1.4%)
1	215 (63.0%)	166 (62.2%)	49 (66.2%)
2	94 (27.6%)	72 (27.0%)	22 (29.7%)
3	21 (6.2%)	19 (7.1%)	2 (2.7%)
More than 3	4 (1.2%)	4 (1.5%)	0 (0.0%)
Number of practitioners within a practice			
1	1 (0.3%)	1 (0.4%)	0 (0.0%)
2	105 (30.8%)	91 (34.1%)	14 (18.9%)
3	99 (29.0%)	87 (32.6%)	12 (16.2%)
4	62 (18.2%)	53 (19.6%)	9 (12.2%)
5	34 (10.0%)	19 (7.1%)	15 (20.3%)
More than 5	40 (11.7%)	16 (6.0%)	24 (32.4%)
Practitioner type within a practice			
Myotherapist	87 (25.5%)	65 (24.3%)	22 (29.7%)
Medical/GP	24 (7.0%)	22 (8.2%)	2 (2.7%)
Medical specialist	15 (4.4%)	10 (3.7%)	5 (6.8%)
Exercise physiologist	20 (5.9%)	15 (5.6%)	5 (6.8%)
Chiropractor	8 (2.3%)	0 (0.0%)	8 (10.8%)
Osteopath	10 (2.9%)	10 (3.7%)	0 (0.0%)
Physiotherapist	49 (14.4%)	39 (14.6%)	10 (13.5%)
Other	205 (60.1%)	158 (46.3%)	47 (63.5%)

ACT-Australian Capital Territory, NSW-New South Wales, NT-Northern Territory, QLD-Queensland, SA-South Australia, TAS-Tasmania, VIC-Victoria, WA-Western Australia. GP-General Practitioner

### Use of superficial heat in the management of LBP

**Fig. 1:**
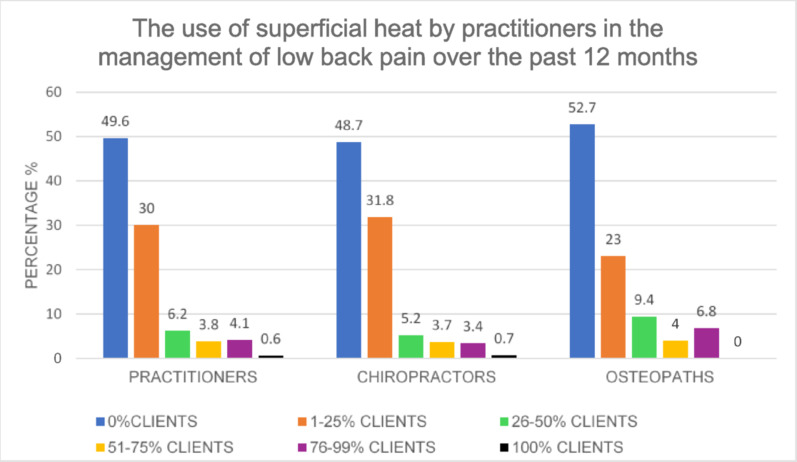
The use of superficial heat by practitioners in the management of low back pain over the past 12 months

Overall patterns of superficial heat use in the management of LBP are shown in Fig. 1. Across the total sample, 44.6% of practitioner respondents (43.2% of osteopaths and 44.9% of chiropractors) reported using superficial heat for the management of LBP in the preceding 12 months. For each profession, a higher percentage of chiropractors than osteopaths reported using superficial heat for the management of chronic LBP (44.6% and 20.3%, respectively). Conversely, a higher percentage of osteopaths than chiropractors reported using superficial heat for the management of acute LBP (20.3% and 12.0%, respectively. The percentage of chiropractors (34.1%) who advised the use of superficial heat for the self-management of LBP was higher than that of osteopaths (28.4%).

### Patient characteristics considered when using superficial heat

**Fig. 2:**
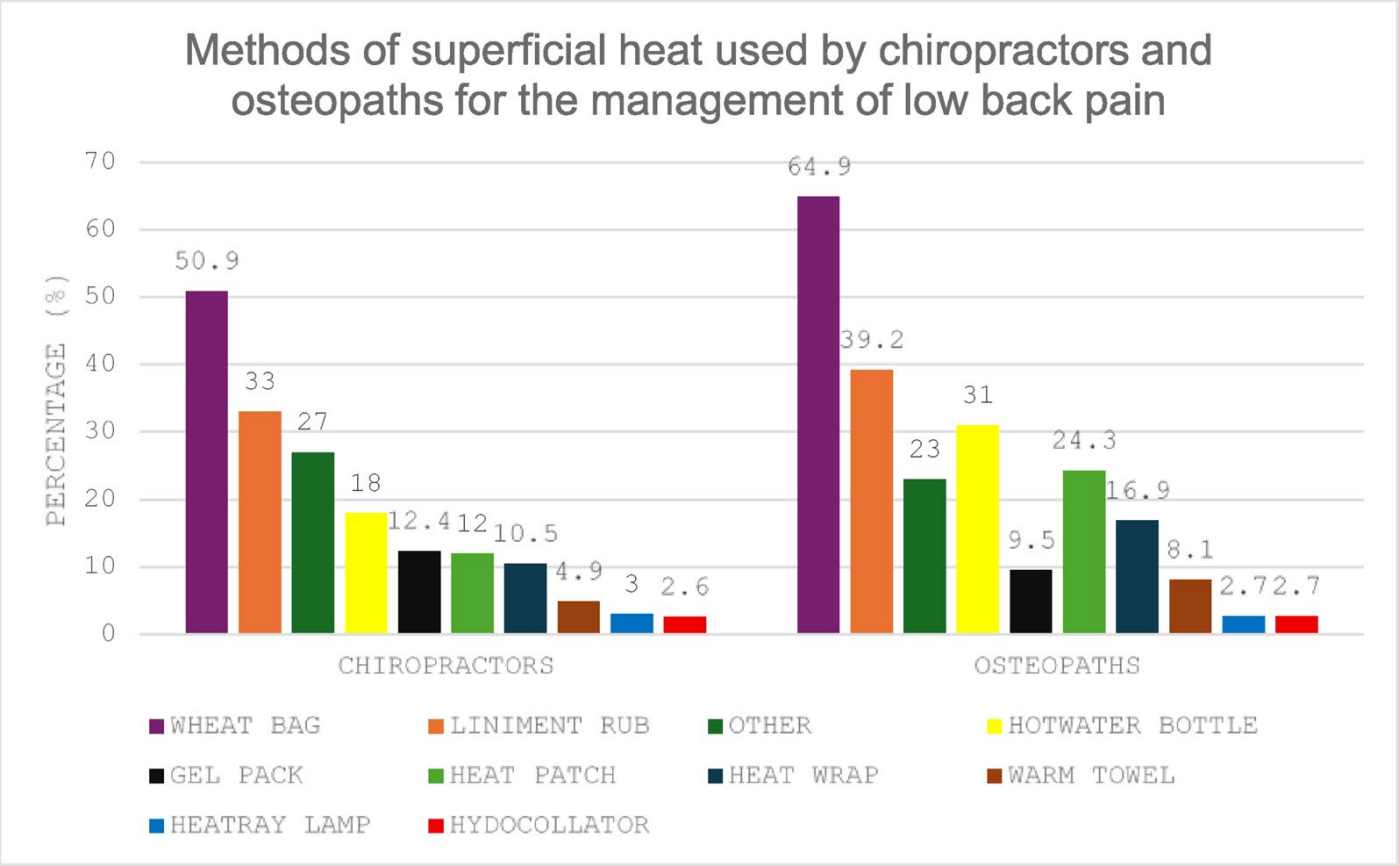
Methods of superficial heat used by chiropractors and osteopaths in the management of low back pain

Chiropractor and osteopath practice characteristics are presented in Table 3. Chiropractors and osteopaths reported that, when superficial heat was applied in the management of low back pain, it was used more frequently in females (29.9%), in cases of non-specific low back pain (27.3%), and in lumbar muscle or myofascial presentations (25.8%). The use of superficial heat differed according to the duration of the patient’s LBP presentation. Respondents reported using superficial heat more frequently for chronic LBP (39.3%) than for sub-acute (11.7%) and acute LBP (13.8%). More chiropractors (44.6%) were likely to preferentially use superficial heat for chronic than for sub-acute (10.1%) or acute presentations (12.0%), while osteopaths were applying heat almost equally to chronic (20.3%), sub-acute (17.6%) and acute (20.3%) of their LBP patients. The preferred methods of application of superficial heat are presented in Fig. 2. Approximately half of the respondents (54.0%) reported a wheat bag as their preferred method for applying superficial heat.

**Table 3:** Typical patient characteristics when practitioners use superficial heat in the management of low back pain

Typical patient characteristics			
	Total sample *n* = 341	Chiropractors *n* = 267	Osteopaths *n* = 74
Gender			
Male	69 (20.2%)	57 (21.3%)	12 (16.2%)
Female	102 (29.9%)	77 (28.8%)	25 (33.8%)
Other (no typical gender)	118 (34.6%)	97 (36.3%)	21 (28.4%)
No response	52 (15.2%)	36 (13.5%)	16 (21.6%)
Age range			
0–17 years of age	5 (1.5%)	4 (1.5%)	1 (1.4%)
18–29 years of age	17 (5.0%)	13 (4.9%)	4 (5.4%)
30–60 years of age	174 (51.0%)	140 (52.4%)	34 (46.0%)
> 60 years of age	64 (18.8%)	60 (22.5%)	5 (6.8%)
No typical age	92 (27.0%)	71 (26.6%)	21 (28.4%)
No response	52 (15.2%)	36 (13.5%)	16 (21.6%)
Lumbar diagnoses			
Lumbar disc	18 (5.3%)	16 (6.0%)	2 (2.7%)
Lumbar facet	28 (8.2%)	18 (6.7%)	10 (13.5%)
Lumbar muscle/myofascial	88 (25.8%)	75 (28.1%)	13 (17.6%)
Non-specific low back pain	93 (27.3%)	71 (26.6%)	22 (29.7%)
Sacro-iliac joint	10 (2.9%)	8 (3.0%)	2 (2.7%)
No specific diagnosis	52 (15.2%)	43 (16.1%)	9 (12.2%)
No response	52 (15.2%)	36 (13.5%)	16 (21.6%)
Low back pain category			
Acute low back pain	47 (13.8%)	32 (12.0%)	15 (20.3%)
Sub-acute low back pain	40 (11.7%)	27 (10.1%)	13 (17.6%)
Chronic low back pain	134 (39.3%)	119 (44.6%)	15 (20.3%)
No typical category	51 (15.0%)	40 (15.0%)	11 (14.9%)
Other	17 (5.0%)	13 (4.9%)	4 (5.4%)
No response	52 (15.2%)	36 (13.5%)	16 (21.6%)
Typical pain referral pattern			
Ankle and foot	1 (0.3%)	1 (0.4%)	0 (0.0%)
Buttock and leg - proximal to knee	117 (34.3%)	90 (33.7%)	27 (36.5%)
Distal leg and ankle	1 (0.3%)	1 (0.4%)	0 (0.0%)
Leg - distal to knee	1 (0.3%)	1 (0.4%)	0 (0.0%)
No typical pain referral pattern	143 (42.0%)	116 (43.4%)	27 (36.5%)
Other	26 (7.6%)	22 (8.2%)	4 (5.4%)
No response	52 (15.2%)	36 (13.5%)	16 (21.6%)

Participants could select multiple responses for the age range item in the questionnaire; therefore, percentages do not sum to 100%

### Awareness, knowledge and use of clinical practice guidelines

Respondents reported consulting a range of clinical practice guidelines to inform their management of LBP, as shown in Table 4. The most frequently used clinical practice guidelines differed between professions. Chiropractors (21.7%) were most commonly guided by the American College of Physicians (ACP), whereas osteopaths (25.7%) mostly referred to the UK National Institute for Health and Care Excellence (NICE UK). Overall, 58.4% of respondents claimed knowledge of how and where to find clinical practice guidelines for LBP, with the highest preferences for the ACP [22] and NICE UK [37] guidelines.

**Table 4:** Clinical practice guidelines used by the total sample, and for chiropractors and osteopaths separately, to inform the management of low back pain

Guidelines used to inform low back pain management			
	Total sample *n* = 341	Chiropractors *n* = 267	Osteopaths *n* = 74
American College of Physicians 2017	67 (19.6%)	58 (21.7%)	9 (12.2%)
American College of Occupational and Environmental Medicine 2019	5 (1.5%)	5 (1.9%)	0 (0.0%)
Belgium Healthcare Knowledge Centre 2017	3 (0.9%)	3 (1.1%)	0 (0.0%)
Danish Health Authority 2017	26 (7.6%)	26 (9.7%)	0 (0.0%)
Canada Institute of Health Economics Towards Optimised Practice 2017	7 (2.1%)	6 (2.2%)	1 (1.4%)
National Institute for Health and Care Excellence-UK 2016	53 (15.5%)	34 (12.7%)	19 (25.7%)
New South Wales Agency for Clinical innovation 2016	29 (8.5%)	21 (7.9%)	8 (10.8%)
Veteran Affairs/ Department of Defense - USA	8 (2.3%)	6 (2.2%)	2 (2.7%)
German Disease Management Guideline Group 2017	2 (0.6%)	2 (0.7%)	0 (0.0%)
Scottish Intercollegiate Guidelines Network UK 2016	3 (0.9%)	2 (0.7%)	1 (1.4%)
None of the above guidelines	57 (16.7%)	47 (17.6%)	10 (13.5%)

ACP-American College of Physicians, ACOEM- American college of occupational and environment medicine USA, Belgium HKC-Belgium healthcare knowledge centre, Danish HA-Danish health authority, Canada IHETOP- Institute of health economics toward optimised practice, NICE UK-National institute for health and care excellence UK, NSW ACI-New South Wales agency for clinical innovation. Australia, Veteran Affairs DD USA-Veteran affairs / Department of defense USA, German Dx Management Group-German disease management guideline group, Scottish Ign UK- Scottish intercollegiate guidelines network UK

## Discussion

This cross-sectional survey provides preliminary national-level insight into chiropractors’ and osteopaths’ reported use of superficial heat for LBP in Australia, including perceived effectiveness and awareness of clinical practice guidelines. Although superficial heat is recognised as a component of multimodal LBP care, its reported clinical use and recommendation for patient self-management were limited in this sample. Chiropractors and osteopaths reported using superficial heat as an adjunct to spinal manipulation in LBP management [25, 29]. However, fewer than half reported use within the preceding 12 months, indicating a potential evidence–practice gap. Importantly, this gap appears to relate not only to whether superficial heat is used, but to how it is delivered, with evidence-supported modalities underutilised in practice. This pattern suggests that awareness alone may be insufficient to drive implementation and aligns with prior evidence demonstrating inconsistent uptake of guideline-endorsed, non-pharmacological interventions across musculoskeletal professions [38, 39].

Clinical practice guidelines, including those of the American College of Physicians, recommend superficial heat as one of several first-line non-pharmacological options for LBP [22]. Within this context, profession-specific differences in reported use were evident: chiropractors reported greater use of superficial heat in chronic LBP, whereas osteopaths were more likely to use it in acute presentations. As guidelines more commonly support superficial heat for acute rather than chronic LBP, these profession-specific patterns suggest possible misalignment between guideline recommendations and reported practice [19, 22, 40].

Superficial heat use was most frequently reported for non-specific and myofascial LBP. This pattern may be partially explained by clinical features commonly associated with midlife and older age, including reduced flexibility and degenerative change of the lumbar spine [41, 42]. In addition to its safety profile [20], superficial heat is thought to influence local circulation and tissue extensibility [23, 43], supporting its use in these presentations. A further observation was that clinical or perceptual factors may influence higher reported use among females; however, this interpretation remains speculative, as these factors were not directly assessed [44, 45].

The mode of application may be a key determinant of both clinical uptake and therapeutic utility. The supporting evidence largely derives from studies of continuous low-level heat wraps that allow mobility, rather than passive modalities such as wheat bags or hot water bottles [20, 24, 46, 47]. Although wheat bags were most commonly reported, their use may limit mobility during application and carry a risk of overheating [48]. In contrast, and more consistent with the available evidence, continuous low-level heat wraps have been associated with improved pain and function while allowing movement [24, 46, 47, 49, 50]. Despite this evidence base, reported uptake in the present study was low (10.5% of chiropractors and 16.2% of osteopaths), further highlighting a discrepancy between the evidence base and reported practice. The Australian Clinical Care Standard for Low Back Pain, which recommends heat wraps as a first-line treatment option [19], provides a relevant national context for these findings. Together, these findings identify an opportunity to better align evidence-supported modes of superficial heat application with clinical practice in Australia.

Despite stronger guideline and trial support for acute presentations, practitioners in our study reported limited use of superficial heat in acute LBP. This contrasts with existing evidence, including a Cochrane review reporting moderate but short-term reductions in pain and disability associated with superficial heat in acute LBP [20]. Freiwald et al. similarly described continuous low-level heat as a safe and practical self-management option [23]. Taken together, these data support superficial heat as a low-risk adjunct within non-pharmacological care pathways, particularly in acute LBP [20].

Variability in practitioner awareness, access, and use of clinical practice guidelines for LBP was also evident. Although approximately half of respondents reported knowing how to access guidelines, only a minority reported applying them in clinical decision-making. Chiropractors most frequently cited the American College of Physicians guideline [22], whereas osteopaths more commonly refer to the UK NICE guideline [37]. This divergence in guideline preference can plausibly map onto different beliefs about when superficial heat fits best. Moreover, consistent with prior literature, barriers such as accessibility, perceived relevance, and limited implementation support may impede uptake [13, 38, 39]. Consolidated repositories and decision aids may facilitate translation into practice.

Recommendations regarding superficial heat are not fully consistent across clinical practice guidelines [12, 14], which may contribute to observed variability in use. Unclear guidance regarding modality and delivery may create uncertainty, and awareness of guidelines does not necessarily translate into consistent application in practice [51]. Unmeasured factors, such as the extent to which guideline recommendations are emphasised during undergraduate training, may also influence practice behaviour. Targeted educational initiatives may therefore help bridge the knowledge–practice gap [52, 53].

Improving implementation will require coordinated educational, behavioural, and system-level strategies that address barriers across practitioner, patient, and system domains, including limited training, time constraints, and concerns regarding safety. Notably, the available evidence supports the safety and cost-effectiveness of superficial heat when appropriately applied [20, 27, 54], suggesting that these barriers may be modifiable. In addition, patient preferences may influence whether superficial heat is recommended or adopted in practice [55]. Addressing these barriers will likely require targeted educational and knowledge translation strategies.

Strengthening undergraduate and postgraduate curricula, along with continuing professional development, could improve practitioner confidence and promote more appropriate clinical use. At a systems level, professional associations, including ACA, CA, OA, are well positioned to disseminate concise, guideline-based resources that emphasise evidence-supported modes of delivery. Collectively, these strategies may support more consistent implementation of guideline-concordant LBP management.

Beyond these implementation considerations, pain mechanisms and local inflammatory processes may influence responsiveness to superficial heat; however, these constructs were not measured in this study and therefore remain speculative [23, 56–58]. Local inflammatory status may provide an additional consideration in understanding variation in response across different LBP presentations. Although pain is commonly classified according to underlying mechanisms such as nociceptive, neuropathic, and nociplastic pain [56], these frameworks may not fully capture the local biological environment of affected tissues, including inflammatory processes [58]. Further research could examine whether integrating inflammatory markers with pain classification better informs the clinical application of superficial heat.

Several limitations should be considered. The cross-sectional design and non-probability convenience sampling limit generalisability and preclude causal inference, and findings should therefore be interpreted as descriptive and exploratory. Reliance on self-reported data over a 12-month recall period introduces potential recall and non-response bias, while volunteer bias may have led to overrepresentation of practitioners with a particular interest in superficial heat. The relatively small sample, particularly for osteopaths, and voluntary participation further limit representativeness, and the absence of weighting or sensitivity analyses may have affected robustness. Future studies using larger, probabilistic samples and mixed-methods designs would strengthen external validity and contextual understanding. Despite these limitations, the inclusion of both chiropractic and osteopathic professions provides clinically meaningful insight into when and for whom superficial heat is used in practice, revealing distinct profession-specific patterns that are directly relevant to everyday clinical decision-making. This may help inform strategies aimed at improving alignment between evidence-supported practice and the delivery of superficial heat in LBP management.

## Conclusion

This cross-sectional survey identified a limited and inconsistent use of superficial heat among chiropractors and osteopaths in the management of LBP. Fewer than half of respondents reported using superficial heat, with chiropractors more likely to apply it in chronic LBP and osteopaths in acute LBP cases. Although most practitioners recognised its therapeutic value and role within multimodal LBP management, actual clinical use and patient self-management recommendations were low. The findings reveal a gap between practitioner knowledge and implementation of guideline-concordant non-pharmacological care. Further research is warranted and should examine the barriers influencing the use of superficial heat, determine optimal application parameters, and evaluate the effectiveness of targeted educational interventions. Improved practitioner training and clearer, more consistent clinical guidance may help facilitate evidence-based integration of superficial heat in LBP management within chiropractic and osteopathic practice.

## Electronic Supplementary Material

Below is the link to the electronic supplementary material.

Supplementary Material 1

## Data Availability

The datasets used and/or analysed during the current study are available from the corresponding author on reasonable request.

## Appendix 1 STROBE Statement - Checklist of items that should be included in reports of cross-sectional studies.

Title: The use of superficial heat in the management of low back pain by chiropractors and osteopaths in Australia: An observational cross-sectional survey.

**Table:**

Item No	STROBE Recommendation	Study-specific information (with page/section references)
Title & Abstract	1a. Indicate the study’s design in the title/abstract	Title includes “cross-sectional survey.” (p 1)
	1b. Provide informative summary	Abstract summarises background, objectives, methods, sample (*n* = 341), key findings, and conclusions. (pp 2–3)
Introduction	2. Background/rationale	Establishes the burden of LBP globally/Australia and the rationale for studying superficial heat use in chiropractors & osteopaths. (pp 2–3)
	3. Objectives	To examine how chiropractors and osteopaths in Australia use superficial heat in LBP management, explore perceptions of its role/effectiveness, and assess awareness of guidelines. (p 2)
Methods	4. Study design	Observational cross-sectional survey using an online questionnaire, guided by the pragmatist paradigm. (p 5)
	5. Setting	National survey conducted June–October 2022 among registered Australian practitioners. (p 6)
	6a. Participants	Inclusion: current AHPRA-registered chiropractors & osteopaths practising in Australia. Recruitment via professional associations (ACA, CA, OA) and conferences/social media. (p 6)
Variables	7. Define outcomes/exposures/confounders	Primary = self-reported use of superficial heat for LBP. Secondary = frequency, method, perceived effectiveness, self-management advice, and guideline awareness. (p 7)
Data sources/measurement	8. Sources & assessment methods	43-item Qualtrics survey capturing demographics, practice data, and clinical behaviours. Pilot-tested for face/content validity. (pp 6,7)
Bias	9. Efforts to address bias	Anonymous online survey, duplicate blocking, pilot testing, training in questionnaire clarity; limitations discussed (selection, recall, social-desirability bias). (pp 6, 16)
Study size	10. How size determined	Target sample: all eligible practitioners reached via associations; descriptive survey without a priori power calculation (acknowledged). (p 6)
Quantitative variables	11. Handling & grouping	Frequencies, proportions, and categories (acute/subacute/chronic LBP; age bands). (p 7,8)
Statistical methods	12a. Describe all analyses	Descriptive statistics (counts, %, frequencies) using SPSS v29. (p 8)
	12b. Subgroups/interactions	Results stratified by profession (chiropractors vs. osteopaths) and by LBP duration. (Tables 1, 2, 3 and 4)
	12c. Missing data	Reported per variable in Tables 1 and 4; low non-response rates (1–15%).
	12d. Sampling strategy	Non-probability convenience sample from professional association databases (p 6)
	12e. Sensitivity analyses	None conducted (descriptive analysis study).
Results	13a. Numbers at each stage	Reports *n* = 341 complete datasets used. (p 8 & Fig. 1)
	13b. Reasons for non-participation	Non-response/decline due to time constraints; discussed as selection bias. (p 16)
	13c. Flow diagram	Figure 1. shows practitioner participation and superficial-heat use categories.
Descriptive data	14a. Participant characteristics	Demographics, practice & patient data in Tables 1, 2 and 3.
	14b. Missing data	Noted (≤ 15% non-response). (p 16)
Outcome data	15. Report numbers or summaries	Proportion using superficial heat = ~ 50%; frequencies by LBP type, method, self-management advice (Figs. 1 and 2 & pp 9–11).
Main results	16a. Unadjusted & adjusted estimates	Only descriptive (no adjustment). (pp 8)
	16b. Category boundaries	Defined for age, years in practice, LBP type, and frequency of use (1–25%, 26–50%, etc.). (p 7, 8, Table 1)
	16c. Absolute risk translation	Not applicable (no risk estimation).
Other analyses	17. Subgroup or sensitivity analyses	Comparative analyses between chiropractors & osteopaths; by acute/chronic LBP category. (pp 9–14)
Discussion	18. Key results	Summarised relative to objectives, limited use of superficial heat, profession-specific patterns, evidence-practice gap. (pp 13–15)
	19. Limitations	Discusses sampling bias, recall bias, small osteopath sample, self-report, and generalizability limits. (pp 15,16)
	20. Interpretation	Contextualised with guideline discrepancies, evidence strength, and education needs, cautious conclusions. (pp 15,16)
	21. Generalisability	Limited to Australian chiropractors & osteopaths; small sample size, external validity discussed. (p 16)
Other information	22. Funding	Supported by CQUniversity (ethics approval HREC 0000022481). Funders had no role in design/analysis. (p 17)
